# Increasing perceived hand size improves motor performance in individuals with stroke: a home-based training study

**DOI:** 10.7717/peerj.7114

**Published:** 2019-07-29

**Authors:** Elisabetta Ambron, Steven Jax, Luis Schettino, H. Branch Coslett

**Affiliations:** 1 Neurology, University of Pennsylvania, Philadelphia, PA, United States of America; 2 Perceptual-Motor Control Laboratory, Moss Rehabilitation Research Institute, Elkins Park, PA, United States of America; 3 Neuroscience program, Lafayette College, Easton, PA, United States of America

**Keywords:** Treatment, Stroke rehabilitation, Magnification of vision, Motor abilities, Body representation

## Abstract

**Background:**

Increasing perceived hand size with magnifying lenses improves tactile discrimination and induces changes in action performance. We previously demonstrated that motor skills (tested with grip force, finger tapping, and a reach to grasp tasks) improved when actions were performed with magnified compared to normal vision; twenty-eight percent of 25 participants with stroke exhibited significant improvement on a composite measure of motor performance with magnification as compared to a session without magnification.

**Methods:**

To investigate the potential implications of magnification of vision for motor rehabilitation, we recruited individuals with stroke from the original cohort who exhibited an improvement of at least 10% in grip force and/or finger tapping for a home training protocol. Six individuals with stroke completed a two-week home-based training program in which they performed a range of activities while looking at their hand magnified. Motor skills were measured before, immediately after, and two weeks after the training.

**Results:**

Five of the six participants showed an improvement on motor tasks when tested after the training. In two participants the improvement was evident immediately after the training and persisted in time, while it occurred at two-weeks post-training in the other participants. These results suggest that the magnification of vision is a potential tool for the rehabilitation of post-stroke motor deficits.

## Introduction

Stroke is one of the most common causes of cognitive and motor deficits in the aging population. After an initial rapid improvement, motor performance in individuals with stroke tends to reach a plateau during the chronic phase after which further improvement is difficult and slow (Page, Gater & Bach-y Rita, 2004). Therefore, it is crucial to develop treatments that improve participants’ performance also in the chronic phase.

To date, treatments to improve the recovery of individuals with stroke have primarily focused on exercising the affected hand, sometimes in combination with electrical stimulation of the peripheral (Koesler et al., 2008) or central (Takeuchi et al., 2005 nervous system) robots (Krebs et al., 2008; Lum et al., 2002; Pelton, Van Vliet & Hollands, 2011) have also been used with some success. Perhaps the most well-studied approach is constraint-induced movement therapy (Wolf et al., 2006) (CIMT). Based on the observation that stroke individuals tend to avoid using the affected hand (Lin et al., 2007), during CIMT patients are forced to use the affected hand by constraining the unaffected arm (Kunkel et al., 1999; Lin et al., 2007; Miltner et al., 1999). One of the beneficial aspects of this therapy is that the training can be home-based and the constraint can be used in the execution of daily activities. On the other hand, not all patients respond well to this type of training and the presence of the constraint is uncomfortable for some patients, creating difficulties in maintaining balance and a sense of frustration when executing tasks unimanually. It has been demonstrated that CIMT induces a rapid cortical reorganization in the motor cortex in which the cortical representation of the affected limb is enlarged (Liepert et al., 1998; Liepert et al., 2000). It is possible that other forms of training that enlarge the cortical representation of the affected hand may also improve motor performance in individuals with stroke.

In a recent transcranial magnetic stimulation (TMS) study of neurologically-intact individuals, we demonstrated that increasing the perceived size of the hand using magnifying lenses enhanced the excitability of the motor system (Ambron et al., 2018a; Ambron et al., 2018b). We found that motor evoked potentials (MEPs) elicited from primary motor cortex were larger when the vision of the hand was magnified; this increase extended to areas surrounding the stimulated spot, suggesting that the magnification of vision may induce enlargement of the cortical representation of the hand when participants viewed their magnified hand as compared to when viewing the hand without magnification. These results are consistent with previous reports of positive effects of visual magnification of the arm on tactile discrimination reported in neurologically-intact individuals (Haggard, Taylor-Clarke & Kennett, 2003) as well as individuals with stroke (Serino et al., 2007). Serino et al. (2007) demonstrated that individuals with stroke performed better on a tactile discrimination task when looking at their hand as compared to when looking at an object contiguous to their hand.

In light of these data, we predicted that magnification of the visual image of the hand of individuals with stroke would improve motor performance. We tested this hypothesis in a pilot study with a convenience sample of twenty-five individuals with chronic stroke (stroke onset >6 months); participants differed substantially with respect to lesion size and location as well as severity of hemiparesis. Participants were tested in 2 sessions; in one session they performed a series of tasks (grip force, finger tapping, reach to grasp, and finger identification) while wearing magnifying lenses, whereas in another session they wore the same visor but with no lens. In both sessions, participants were also tested in a second block of the same tasks approximately 10 min later to assess the persistence of any observed effects (Ambron et al., 2018a; Ambron et al., 2018b). Twenty-eight percent of participants demonstrated significantly better performance with magnification on a composite measure of motor performance; the effect persisted over a brief interval of 30–60 min in the post-magnification block. To the best of our knowledge, this represents the first report of an effect of magnification on motor performance in participants with brain lesions.

We regarded these results as promising and undertook the study reported here to determine if magnification of vision could be a useful tool for rehabilitation of motor functions in stroke individuals. To that end, we recruited participants from our pilot study who had demonstrated at least 10% improvement in performance with magnifying lenses on grip strength or finger tapping tasks to participate in a two week study in which participants used magnifying lenses daily for one hour while performing activities such as drawing or assembling a puzzle. Motor performance was measured before, immediately after, and two weeks after the end of training. As the study was regarded as a preliminary, proof of principle investigation exploring a novel intervention about which little is known, we conducted a small study designed to detect a treatment effect that, if identified, would be systematically explored in subsequent work. To that end, we did not include a control group and included only participants who, in a single session, had previously demonstrated benefit from magnification.

## Material and Methods

Participants were recruited from the cohort of 25 individuals with hemiparesis from chronic stroke who participated in the previous study of the effects of magnification on motor performance^18^. Participants for the present study were selected because they showed an improvement in the grip force and/or finger-tapping task of at least 10% in the session with magnifying glasses as compared to normal vision. We focused on these two tasks, as they are known to be predictors of stroke recovery (Boissy et al., 1999; Mercierand & Bourbonnais, 2004; Sunderland et al., 1989). Eleven individuals with stroke met entry criteria; eight agreed to participate but only six completed the training (see Table 1). Two individuals initially agreed to participate but acknowledged not having performed the training, because they forgot or did not have time.

**Table 1:** Demographics, brain lesions information, percentage of improvement with magnified vision observed in a previous study (Ambron et al., 2018a; Ambron et al., 2018b) in Grip force and Finger tapping, and Fugl-Meyer score of individuals recruited in the present study.

**Numb**	**Sex**	**Age**	**Education**	**Handedness**	**Side**	**Lesion volume (cc)**	**Lesion site**	**Hand used**	**Fugl- Meyer**
**1**	M	74	13	R	LBD	33.09	Basal ganglia, post. Limb of IC	Contralesional	51
2	F	67	12	R	LBD	1.87	Basal ganglia; post. Limb of IC	Contralesional	47
4	F	53	13	R	RBD	67.7	Fronto-temporal, centrum semiovale	Contralesional	20
**5**	M	69	11	R	RBD	N.A.	Fronto-parietal	Contralesional	41
**6**	F	53	16	R	LBD	N.A.	Cerebellum	Ipsilesional	N.A.
8	F	70	13	R	LBD	N.A.	Internal capsule	Contralesional	44

**Notes.**

N.A not available IC inferior colliculus

Demographic characteristics and information regarding brain lesions of the final sample of six participants are presented in Table 1. The sample included four left brain-damaged (LBD) and two right brain-damaged (RBD) individuals. All participants used the affected arm (contralesional in five and ipsilesional in one participant with cerebellar lesion). Participants had upper extremity Fugl-Meyer scores (Fugl-Meyer et al., 1975) ranging from 20 to 51.

Participants were presented with a battery of tasks assessing motor and somatosensory abilities on three different occasions: before the training (PRE condition), immediately after the completion of 2 weeks of training (end of training), and two weeks after the completion of training (2-week follow-up). This battery included the Action Research arm test (ARAT) (Yozbatiran, Der-Yeghiaian & Cramer, 2008), the Rivermead Assessment of Somatosensory Performance (RASP), maximum grip strength, finger tapping, and a reach to grasp task used in a previous study (Ambron et al., 2018a; Ambron et al., 2018b). The ARAT is a small battery measuring the ability to perform different movements (to grasp, to grip, to pinch and to perform gross movements). During the RASP, participants were tested on sensory extinction, two-point discrimination, and the upper limb proprioception movement and direction discrimination tests. In the sensory extinction task participants were asked to identify whether the examiner stimulated the left, right or both hands (or cheeks) (maximum score of 12). In the two-point discrimination, participants were asked to identify whether their index finger was stimulated with one or two points (score as *failed* or *passed*). Finally, proprioception was assessed by asking participants to identify the direction of the movement (up or down) that the examiner made to the participant’s elbow, wrist, or thumb (maximum score of 30). All the assessments were conducted in a laboratory of Moss Rehabilitation Research Institute or the University of Pennsylvania.

For the grip strength task, participants were asked to squeeze a digital handgrip dynamometer (CAMRY 90 kgs) while looking at their hand; for each of the 6 trials, the maximum grip strength in kg was recorded. Finger tapping was assessed by asking participants to press and release a telegraph key (Electronic Tapping Test, Western Psychological Services) with their index finger as often as possible in 10 s interval while looking at their hand. The total number of finger taps was recorded for each of the trials.

Finally, in the reach to grasp task, participants were required to reach and grasp wooden geometrical shapes, including a sphere, a parallelepiped, and a pentagonal shape with a triangular protrusion extending on the high edge (convex shape). Objects were ∼7 cm in height, ∼9.5 cm in length, and ∼2.8 cm in width and weighted average of 80 g. Objects were presented on a square dowel rod mounted on a plastic stand (see Ambron et al., 2017). Participants performed 30 trials (six with the sphere; six with the parallelepiped and 6 with the convex shape presented in horizontal orientation and 6 with the parallelepiped and 6 with the convex shape presented in vertical orientation). A 3D electromagnetic tracking system (trackSTAR, Ascension Technologies Inc) with 6DOF tracking sensors at a sampling rate of 100 Hz and a custom program written in C++ were used to record participants’ movements. We used one sensor at the center of the dorsal part of participants’ wrist to extract the movement time (MT), using custom Matlab routines. Movement onset and offset were defined as the times required to reach 5% of peak velocity and to decrease to below 5% of peak velocity.

**Figure 1:**
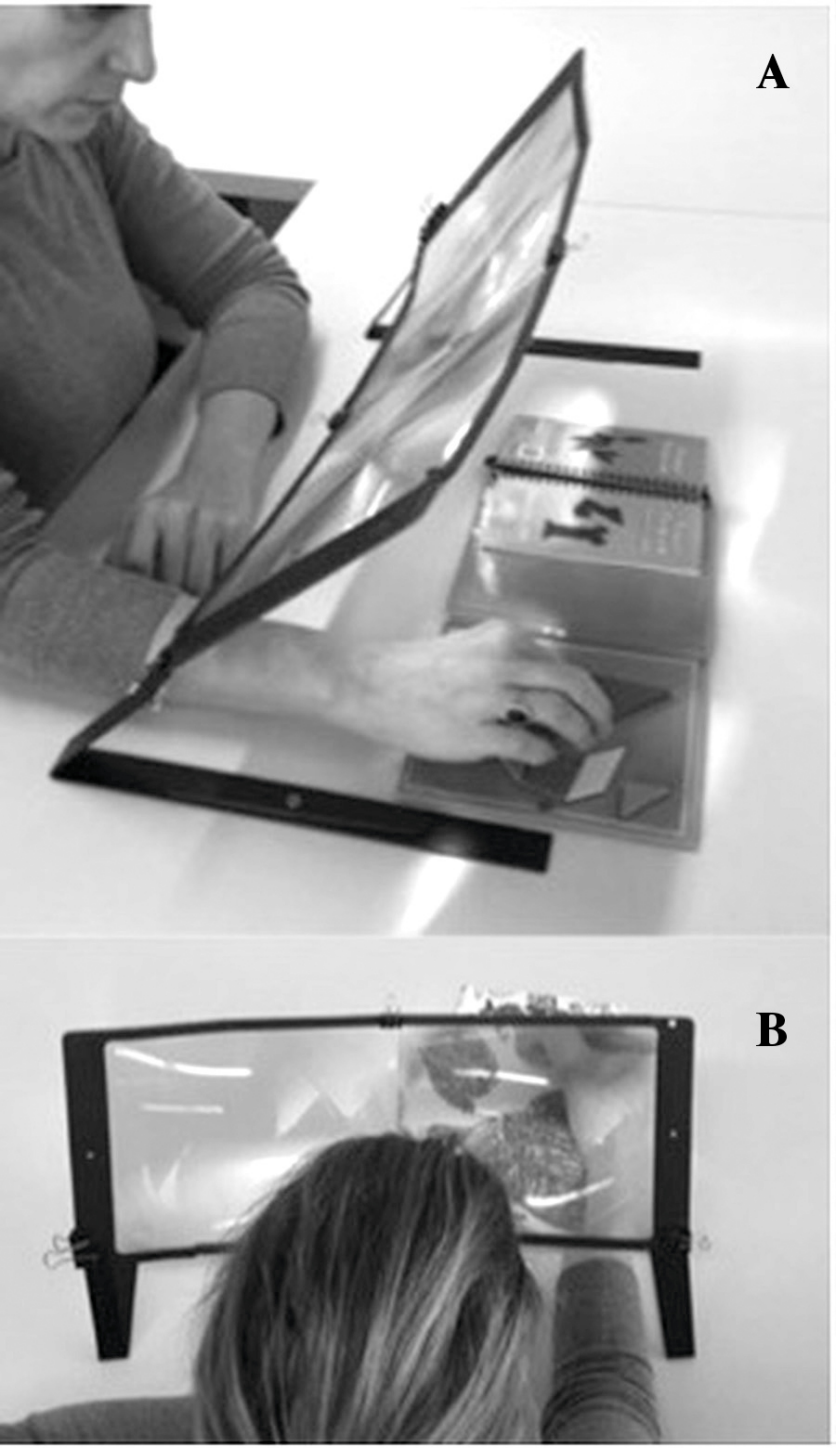
Different views of the two hand Free Page Magnifiers set up used during the training.

The training consisted of 30-minute daily sessions for two weeks in which participants performed a series of tasks (e.g., assembled puzzles, coloring drawings) while viewing a magnified image of their hand(s). Participants’ hand(s) and viewed objects were magnified by a factor of 2 by looking through two Hands-Free Page Magnifiers (8′ × 22′; Prop-It) magnifying lenses that were placed on the table on which they worked (Fig. 1). Participants (or caregiver when needed) set up the magnifiers on a tabletop and were instructed to use the affected arm and, if the activity required, their unaffected hand; participants (or caregiver when needed) videotaped the training using a small camera placed on the table. Participants’ feedback after the training was positive: they enjoyed the activities and did not find the training particularly demanding. All sessions were self-administered and performed at home at a time chosen by the participant. All participants signed an informed consent prior to starting the experiment and the study was approved by the institutional review board (IRB) (reference number 702940) of the University of Pennsylvania and the Einstein Healthcare Network. This study was registered at ClinicalTrials.gov (number: NCT02898558).

## Results

Five of 6 participants demonstrated a beneficial effect of training on one or more tasks in the comparison across all three testing times (Table 2). Two participants showed significant improvement in the comparison between PRE and the end of the training, and five participants showed significant improvement in 2-week follow-up compared to PRE. Performance at 2-week follow-up was better than at the end of the training on at least one measure in 4 participants.

**Table 2:** Patients scores in the average score in the ARAT (impaired hand) grip force, finger tapping, and in movement time of the reaching and grasping task. In parentheses the percentage of improvement with respect to the pre condition, and Friedman test results in the comparison across testing sessions. Percentages were not reported if an improvement between sessions was not observed.

**Subject code**	**Task**	**Pre**	**End of the training**	**2-weeks follow-up**	**Friedman test**
**1**	ARAT	25	34	35	
	GRIP FORCE	17.3*#	20.5 (19%)*$\hat {}$	19.2 (11%)#$\hat {}$	**X**^**2**^**(2) = 10.33**, ***p* = 0.006**
	FINGER TAPPING	34.1	34.6	32.1	X^2^ (2)=.78, *p* = 0.67
	REACH TO GRASP	2481.15#	2347.50 (5%)$\hat {}$	2033.08 (18%)#$\hat {}$	**X**^**2**^**(2) = 22.1**, ***p***<**0.001**
2	ARAT	47	47	48	
	GRIP FORCE	19.2*	17.9*	17.9	X^2^(2) = 6.33, *p* = 0.04
	FINGER TAPPING	55	56.3 (2%)	56.6 (8%)	X^2^(2) = 3.7, *p* = 0.15
	REACH TO GRASP	2189.60*#	1937.78 (11%)*	1890.0 (14%)#	**X**^**2**^**(2) = 12.9**, ***p* = 0.002**
4	ARAT	34	38	24	
	GRIP FORCE	5	5	5.7 (14%)	X^2^(2) = 1.6, *p* = 0.43
	FINGER TAPPING	22.8	19	23.1 (14%)	X^2^(2) = 1.6, *p* = 0.43
	REACH TO GRASP	2187.08*#	2882.41*$\hat {}$	2445.71#$\hat {}$	X^2^(2) = 33.3, *p* <0.001
**5**	ARAT	42	43	39	
	GRIP FORCE	9.6#	11.5 (19%)$\hat {}$	13.5 (38%)#$\hat {}$	**X**^**2**^**(2) = 10.33**, ***p* = 0.006**
	FINGER TAPPING	38.5#	34.3$\hat {}$	45.6 (19%)#$\hat {}$	**X**^**2**^**(2) = 7**, ***p* = 0.03**
	REACH TO GRASP	CNT			
**6**	ARAT	48	48	48	
	GRIP FORCE	19.4#	20.6 (6%)$\hat {}$	21.7 (12%)#$\hat {}$	**X**^**2**^**(2) = 9.6**, ***p* = 0.008**
	FINGER TAPPING	28.6#	33.6 (17%)$\hat {}$	50.8 (77%)#$\hat {}$	**X**^**2**^**(2) = 10.33**, ***p* = 0.006**
	REACH TO GRASP	2345.71#	2342.14$\hat {}$	2082.41 (11%)#$\hat {}$	**X**^**2**^**(2) = 19.7**, ***p***<**0.001**
8	ARAT	48	48	48	
	GRIP FORCE	12.7*#	9.9*$\hat {}$	16.6 (30%)#$\hat {}$	**X**^**2**^**(2) = 8.3**, ***p* = 0.02**
	FINGER TAPPING	40.6	41.1 (2%)	39.6	X^2^(2) = 4.9, *p* = 0.08
	REACH TO GRASP	2005.83	2049.29(2%)	1801.07(10%)	X^2^(2) = 5.1, *p* = 0.07

**Notes.**

Significant differences (*z*>2.0; *p*<0.05) obtained with Wilcoxon signed-rank test in the comparison between: * PRE and POST 1; # PRE and POST 2; $\hat {}$ POST1 and POST 2.

N.E.: not executed for safety reasons as SUB 5 had a pacemaker.

The tasks yielded somewhat different findings. For the grip force task, Friedman test was significant in 4/6 participants: one participant (SUB1) showed an improvement at the end of the training, four participants (SUB1,5,6,8) at 2-week follow-up as compared to PRE, and three participants at 2-week follow-up as compared to the end of the training.

Two participants (SUB5,6) demonstrated a significant improvement on the finger tapping task: their performance was better at 2-week follow-up than both PRE and the end of the training.

Three participants demonstrated significant reduction in MT on the reach-to-grasp task: SUB2 improved at both the end of the training and 2-week follow-up, whereas SUB1 and 6 demonstrated significant improvement at 2-week follow-up relative to PRE. Finally, SUB4 showed longer MT at the end of the training and 2-week follow-up relative to PRE.

Most participants performed well in the ARAT across sessions and at ceiling in the RASP; SUB4, the most impaired participant at Fugl-Myer, scored 9, 10 and 10 on the ARAT across sessions.

## Discussion

We report for the first time that subjects with motor deficits from stroke may demonstrate significant improvement in motor function after 2 weeks of daily 30-minute sessions during which they performed manual activities while gazing through a magnifying lens. From the previous cohort of twenty-five participants, 11 out of 25 (44%) participants showed at least 10% improvement in a motor task with the magnification of vision and were recruited for the present study. Of these, six participants completed the training and five demonstrated significant improvement in motor functions after 2 weeks of daily 30-minute sessions during which they performed motor tasks while gazing through a magnifying lens. Of the five participants who showed a beneficial effect of magnification, two participants showed an effect immediately after the training while all five participants showed an improvement at 2-weeks follow-up compared to baseline, and also at 2 weeks follow-up as compared to end of training.

Our findings confirm and extend our previous reports of beneficent effects of magnification of vision for motor function in normal (Ambron et al., 2017; Ambron et al., 2018a; Ambron et al., 2018b) and brain lesion (Ambron et al., 2018a; Ambron et al., 2018b) subjects. In particular, these data extend our previous report of benefit from a single session of magnification in subjects with stroke in several important respects. First, the previous study (Ambron et al., 2018a; Ambron et al., 2018b) contrasted the effect of magnification to normal vision, each assessed in separate sessions. Here we report data from a comparison of normal vision to normal vision, assessed before and after daily training with magnification daily over a two-week interval; thus, the benefits reported here are unlikely to reflect non-specific effects of factors such as arousal associated with a novel intervention as the data were collected with participants in their usual (no magnification of vision) state. Second, in our previous work with a single session of magnification of vision, we assessed carry-over of benefits after only 20 min. The current study demonstrates that beneficial effects persisted and increased over the 2-week follow-up period; as will be discussed below, this finding raises the possibility that repeated intervals of magnification of vision fundamentally alters the representation of the participant’s motor system. In light of these considerations, we believe these data to be promising as they raise the possibility that the simple magnification of vision could be used as a tool for home-based rehabilitation training.

These data suggest that repeated intervals of magnification of vision fundamentally alters subjects’ sensori-motor representations for the affected hand; we speculate that this is attributable to an increase in motor cortex excitability or enlargement of the cortical area subserving the magnified hand. We suggest that, as in normal participants (Ambron et al., 2018a; Ambron et al., 2018b) magnifying the image of the participant’s hand induced plasticity in the participant’s motor system. Thus, participants with stroke may have experienced an increase in cortical excitability as well as in increase in the cortical region subserving the hand while it was magnified; whereas a single session of magnification may have induced a state-dependent change, repeated exposures may have induced a lasting alteration in the cortical representation of the motor system. The claim that improvement in motor function may be associated with cortical remapping is, of course, not without precedent (Liepert et al., 2000). We note that because improvement was maximal at the time of our last assessment, it is possible that participants continued to improve and that our data underestimate the beneficial effects of the intervention.

Another positive feature of our intervention is that it is home-based. Participants performed the training in a comfortable and familiar environment at a time that was convenient for them. Furthermore, the activities to be performed during training were more entertaining than many rehabilitation activities that focus on repetition of a limited repertoire of actions. For both puzzle assembling and drawings, participants were asked to perform several reaching and grasping movements with the impaired hand. Participants had the freedom to choose the activities that they wanted to carry out. Importantly, our intervention is relatively inexpensive, as it does not require the presence of a therapist or expensive equipment.

The study and conclusions drawn from it are subject to substantial limitations. First, given the sample size and lack of a control group, the work constitutes a proof of principal that requires additional investigation with larger, blinded studies. Second, the training regimen was not intensive; previous works using movement constraint therapy used protocols consisting of up to six hours of therapy a day (Kwakkel et al., 2015). The fact that participants employed the magnification system for only 30 min daily may have limited the effectiveness of the intervention. Third, participants were selected on the basis of benefit observed in a single session of magnification, potentially biasing the results. As the relationship between a positive response to a single session of magnification and benefit from a 2-week training protocol has not been studied, the consequences of our recruitment strategy is unclear. Future work should investigate if beneficial effects can also be observed in individuals who do not show an immediate effect of magnification of vision in motor performance. We suggest, however, that even should benefit from magnification only be observed in participants who demonstrated improvement on a screening session, magnification of vision may prove to be a useful therapeutic approach given that 44% of an unselected group of participants demonstrated at least 10% benefit in the screening test; thus, based on the assumption that the treatment protocol benefits only subjects who demonstrated a benefit in the screening session, magnification therapy would have benefited 37% of participants (that is, 11/25 × 5/6). Fourth, the present group of participants included people with stroke at a variety of lesion sites (basal ganglia, frontal, parietal and temporal areas) and size (Table 1). The fact that participants with diverse lesion sites benefited from magnification suggests that the intervention may prove to be beneficial in many participants with stroke. On the other hand, the identification of target populations that can benefit from this training the most is a clinically relevant question, and future work should address the effect of magnification in groups of patients with more selective lesions. Finally, the crude vision magnifying system used in our study substantially limited the scope and type of activities in which participants could engage with magnified vision, thereby limiting interest in the intervention. Future randomized, blinded studies that include participants independent of their baseline performance with magnification employing engaging virtual or augmented reality would address all of these issues.

## Conclusions

To conclude, these preliminary results suggest that home-based training using magnification of vision has the potential to improve motor performance in individuals with stroke.

##  Supplemental Information

10.7717/peerj.7114/supp-1 Supplemental Information 1 Grip and Tapping task Grip and Tapping task trials for every patient across condition.

10.7717/peerj.7114/supp-2 Supplemental Information 2 Raw data Movement Time Movement time trials for every patient across condition.

## Additional Information and Declarations

### Data Availability

The following information was supplied regarding data availability: Raw data is available in the Supplemental Files.
